# Burden of pelvic organ prolapse in Ethiopia: a systematic review and meta-analysis

**DOI:** 10.1186/s12905-020-01039-w

**Published:** 2020-08-06

**Authors:** Getnet Gedefaw, Asmamaw Demis

**Affiliations:** 1 Department of Midwifery, College of Health Sciences, Woldia University, P.O.Box: 400, Woldia, Ethiopia; 2 Department of Nursing, College of Health Sciences, Woldia University, P.O.Box: 400, Woldia, Ethiopia

**Keywords:** Pelvic floor disorders, Systematic review, Meta-analysis

## Abstract

**Background:**

Pelvic organ prolapse can significantly affect a woman’s quality of life by compromising physical, social, psychological and sexual function. Pelvic organ disorders and its consequences have higher economic burden to the patient as well to the country. Therefore, this systematic review and met- analysis aimed to estimate the burden of POP in Ethiopia.

**Methods:**

International databases (MEDLINE/Pub Med, Hinari, Scopus, Google scholar, African journals and literatures were searched and seven eligible cross sectional and two case control studies were included in this systematic review and meta-analysis. Eggers test and funnel plot were computed to check publication bias across the studies. Publication bias was computed using a funnel plot and eggers test. Heterogeneity of the studies was checked using Cochrane Q-test and I^2^ statistic. Subgroup analysis was computed for the evidence of heterogeneity.

**Results:**

This systematic review and meta-analysis revealed that the overall national prevalence of pelvic organ prolapse in Ethiopia was 23.52% (95% CI: 61.04, 80.24). Being rural resident (AOR = 3.29; 95% CI: 1.38–7.85), I^2^ = 47.5%, *P* = 0.167), having < 18.5 BMI (AOR = 2.59; 95% CI: 1.53–4.4), I^2^ = 59.9%, *P* = 0.64), and age > 40(AOR = 7.43; 95% CI: 2.27–24.29), I^2^ = 75.9%, *P* = 0.016) were the associated risk factors for pelvic organ prolapse.

**Conclusions:**

The pooled prevalence of pelvic organ prolapse was high. Residence, body mass index and age of the women were the predictors of pelvic organ prolpase. Creating awareness and identifying the modifiable and non modifiable risk factors for pelvic organ prolpase is a crucial strategy to prevent further complications and risk of operation.

## Background

Pelvic organ prolapse is a complex condition resulting from the weakness and defects of pelvic floor structures. Pelvic organ prolapse (POP) is the descent of one or more aspects of the vagina and uterus: the anterior vaginal wall, posterior vaginal wall, the uterus (cervix), or the apex of the vagina (vaginal vault or cuff scar after hysterectomy) [1].

Worldwide, millions of women have been affected by pelvic floor disorders. One in every nine American women will undergo surgery for a pelvic floor disorder in her lifetime, with 30% of women having a chance of requiring additional surgery for the same condition [2, 3].

Pelvic organ prolapse occurs when the pelvic floor no longer supports the proper positioning of the pelvic organs, i.e. the vagina, bladder, rectum or uterus. In unites states of America, around 6–7% of the women having pelvic floor disorders were experiencing pelvic and urinary symptoms. Feeling of vaginal bulging and/or pelvic pressure, urinary incontinence, and constipation has been identified as symptoms of pelvic organ prolapse [4–6]. In different countries, the number of admissions for POP surgery was significantly increasing; for instance, the rate of admission for POP surgery per 1000 women 0.87, 1.14, 1.13 in Germany, France, and England respectively [7]. The estimated prevalence of pelvic organ prolapse (POP) has been reported with a figure of nearly 9%, however, the burden of POP in low and middle income countries particularly in Sub Saharan African countries is still challenging. Moreover, the prevalence of POP in low income countries is much more increasing which recently has been reported to be around 20% [8, 9].

POP and its complications make an impact on a considerable economic burden on the affected person. About 11% of the American women undergo surgery for POP or incontinence before the age of 79, with 29.2% of the women having a chance of requiring additional surgery [10].

Pelvic organ prolapse is highly prevalent among women with the age of over 40 years old, geriatric women, and postmenopausal women with the estimated prevalence of 41–50% [11, 12].

As different studies, in low and middle income countries showed the mean of pelvic organ prolaps is 19.7% with the estimated range from 3.4 to 56.4%. Moreover, most studies were small and not population based, as a result methods of ascertaining of POP and definitions have been varied [9].

High parity, older age, obesity, pregnancy and vaginal delivery, menopause, constipation, persistent coughing, early age at first delivery, forceps delivery, prolonged second stage labor, and prolonged heavy lifting are the revealed contributing factors to cause strain to the pelvic floor. The global burden of Pelvic floor disorder is increasing due to increasing the age of the population [13].

The symptoms pelvic floor disorders had affect many aspects of a woman’s life, such as social, psychological, occupational, domestic, physical, cognitive, behavioral and sexual domains. In general, pelvic floor disorders have a negative impact on women’s lives, emotional and quality of life. Certainly, POP may be associated with a variety of systemic symptoms such as urinary, bowel and sexual symptoms which may significantly confrontation the quality of life of the women have been facing different mental and psychological illness such as mood and personal disorders [14, 15].

In Ethiopia, institutional delivery is limited, high fertility rates and higher rates of gynecologic disease have been reported and only 48% of women deliver in health institutions [16].

In Ethiopia, there is no any take apart strategy to prevent and manage pelvic floor disorders both in private and governmental health institutions. Even if, different single studies have been tried to identify the types and levels of pelvic floor disorders, there has been limited source of evidence regarding types, and burdens of POP in Ethiopia. As a result, this study helps to estimate the national burden of Pelvic organ prolapse at national level to design appropriate preventions and management modalities.

## Methods

This systematic review and meta-analysis were conducted to estimate the national burden of POP and its associated factors in Ethiopia using the standard PRISMA checklist guideline.

### Searching strategy

International databases (MEDLINE/Pub Med, Hinari, Scopus, Google scholar, African journals and literatures) were searched and included in this study. Searching terms were used using PICO formulating questions. The following Searching terms were used: pelvic floor disorders, POP, utero-vaginal prolapse, vault prolapse, rectocele, cystocele AND Ethiopia and related terms.

### Eligibility criteria

#### Inclusion criteria

##### Study design

Observational studies (cross-sectional, cohort and case-control) were included.

##### Study area

Only studies conducted in Ethiopia without time limiting and reported the prevalence or at least one least adjusted associated factor of pelvic organ prolapse were included.

##### Publication status and language

Only English language articles both published and unpublished reported studies were included.

#### Exclusion criteria

Articles without full abstracts or texts and articles reported out of the outcome interest were excluded.

### Quality assessment

After collecting the findings from all databases, the articles were exported to Microsoft Excel spreadsheet. Two authors (GG & AD) independently extracted the data and reviewed the screened and eligible articles. Any disagreement was handled by the two reviewers (GG & AD). Finally, a consensus was reached between two authors through discussion. The methodological quality of each study (sampling strategy, response rate, and representativeness of the study), comparability and outcome were checked using the NOS tool. Newcastle-Ottawa Quality Assessment Scale (NOS) for cross sectional and case control studies were used to assess the methodological quality of a study and to determine the extent to which a study has addressed the possibility of bias in its design, conduct and analysis. All of the included articles scored (NOS) 7 and more can be considered as “good” study with low risk (Table S1).

### Measurement of outcome

In this study measurement of outcome was measured and included from studies report POP depending on either **POP-Q stage** or **the traditional classification of POP.**

#### POP-Q stage

- **Stage-0** “no prolapse”
- **Stage I** is when the most distal portion (leading surface) of the prolapse is 1 cm above the level of the hymen (,-1 cm)
- **Stage II** is when the most distal portion (leading edge) of the prolapse is #1 cm proximal to or extends 1 cm through the plane of hymen ($-1 cm, but # + 1 cm)
- **Stage III** is when the most distal portion of the prolapse is .1 cm below hymen but no further than 2 cm less than the TVL (there is no complete vaginal eversion)
  **Stage IV** is when there is complete eversion of the total length of the pelvic organ, meaning the pelvic organ protrudes at least the total vaginal length minus 2 cm beyond the hymen.

#### Traditional classification of POP

- 1st: Descent within the vagina
- 2nd: Descent to the introitus
- 3rd: Descent outside the introitus

##### Pelvic organ prolapse

Any pelvic disorder at least reported any of one of the pelvic floor disorders (utero-vaginal prolapse, rectocele, cystecole, vault prolapse) with any stages were considered as the primary outcome of the study.

##### Anterior compartment prolapse (cystocele)

Hernia of anterior vaginal wall often associated with descent of the bladder.

##### Posterior compartment prolapse (rectocele)

Hernia of the posterior vaginal segment often associated with descent of the rectum.

##### Apical compartment prolapse (uterine prolapse, vaginal vault prolapse)

Descent of the apex of the vagina into the lower vagina, to the hymen, or beyond the vaginal introitus.

### Data extraction

Micro-soft Excel (2016), and STATA version 11 software were used for data entry and analysis respectively. Two authors (AD and GG) independently extracted all the important data using a standardized JBI data extraction format. Substantial agreement between reviewers i.e. Cohen’s kappa coefficient > 0.60 were accepted. Any disagreement between reviewers was resolved through discussion and then consensus was reached. During data extraction; name of the author, sample size, publication year, study design, prevalence, response rate, population outcome, study site, and different contributing factors were included. Moreover, prevalence of pelvic organ prolapse with 95%CI and associated factors were collected [17].

### Data analysis

Egger’s regression test and funnel plot were computed to check the publication bias across and within the studies. Cochrane Q-test and I-squared statistic were used to check the heterogeneity of studies [18]. Pooled analysis was computed using a weighted inverse variance random-effects model [19]. Subgroup analysis was done by study setting, study region and year of publication. The data were analyzed and presented using STATA version 11 statistical software and forest plot respectively. The pooled prevalence of POP was expressed with 95%Cl, where as a log odds ratio was used to declare the association between associated factors and POP [17].

## Results

### Characteristics of the included studies

238 articles were retrieved using a search strategy about pelvic organ prolapse and associated factors in Ethiopia using online international datasets such as; MEDLINE/PubMed, Hinari, Scopus, Google scholar, African journals and literatures. After duplicates were removed, 117 studies remained.

**Fig. 1:**
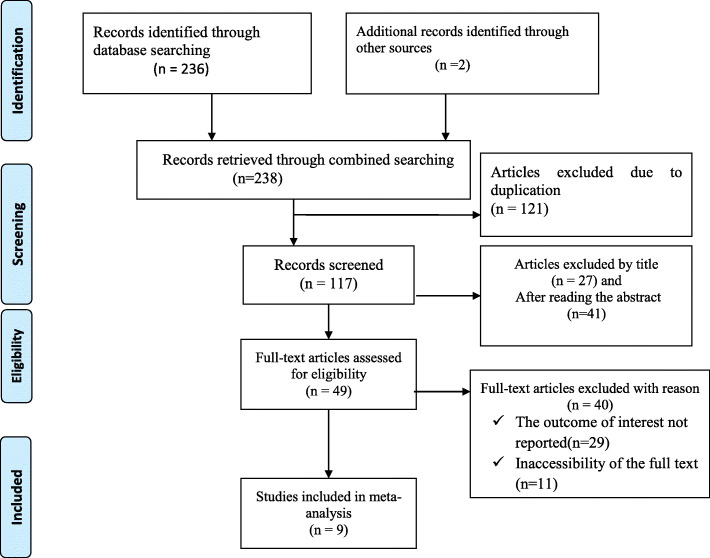
Flow chart of selection for systematic review and meta-analysis on the burden of POP in Ethiopia

68 articles were excluded after review of their titles and abstracts from the remaining 117 articles. Therefore, 49 full-text articles were accessed and assessed for inclusion criteria, resulting further exclusion of 40 articles primarily due to reasons (Fig. 1). As a result, 9 studies were fulfilled the inclusion criteria to undergo the final systematic review and meta-analysis (Table 1).

**Table 1:** Study characteristics included in the systematic review and meta-analysis on the burden of POP in Ethiopia

Authors	Region	Study setting	Study design	Sample size	Prevalence	Quality
Tsegay B [20]	Tigray	Facility based	Cross-sectional	370	NA	Low risk
Asresie et al. [21]	Amhara	Facility based	Case control	1676	42.3	Low risk
Andualem Henok [22]	SNNPR	Facility based	Cross-sectional	422	13.27	Low risk
Berihun et al. [23]	Amhara	community based	Cross-sectional	395	11.9	Low risk
Dheresa et al. [24]	Oromia	Community based	Cross-sectional	3432	20.5	Low risk
*Menur A.* et al [25]	Oromia	Facility based	Cross-sectional	129	40.7	Low risk
Lukman [26]	AA	Facility based	Cross-sectional	195	17.2	Low risk
Lukman [26]	Amhara	Facility based	Cross-sectional	156	19.9	Low risk
Zinash et al. [27]	SNNPR	Facility based	Case control	318	NA	Low risk

### Magnitude of pelvic organ prolapse in Ethiopia

**Fig. 2:**
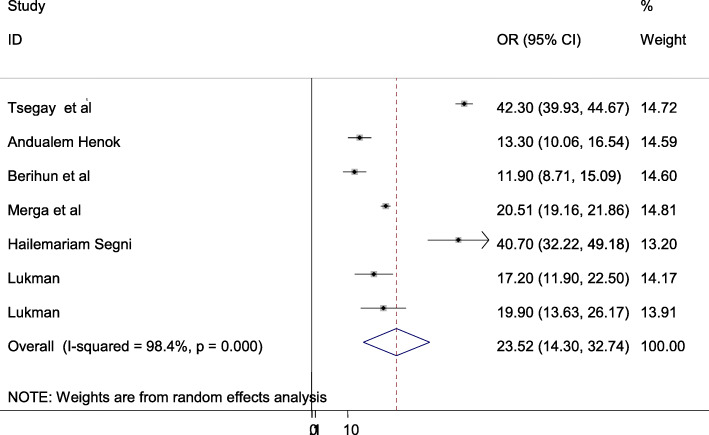
Pooled prevalence of pelvic organ prolapse in Ethiopia

The overall burden of pelvic organ prolapse is presented with a forest plot (Fig. 2). Therefore, the national estimated prevalence of pelvic organ prolapse in Ethiopia was 23.52% (95% CI: 14.3–32.74; I^2^ = 98.4%, *P* < 0.001).

### Publication bias

**Fig. 3:**
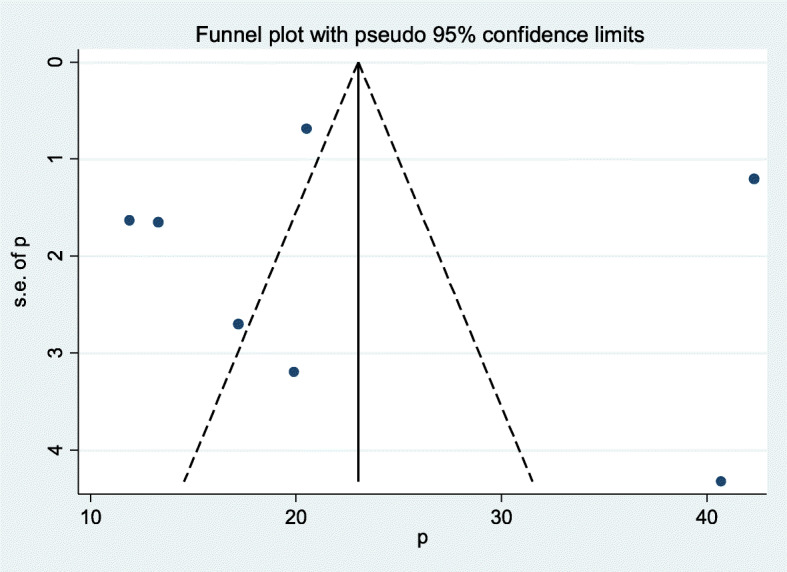
Funnel plot showed publication bias for pelvic organ prolapse

Funnel plot was assessed for asymmetry distribution of pelvic organ prolapse by visual inspection **(**Fig. 3). Egger’s regression test showed a *p*-value of 0.967 with no evidence of publication bias.

### Subgroup analysis

Subgroup analysis was employed with the evidence of heterogeneity. In this study, the Cochrane I^2^ statistic was 98.4%, *P* < 0.001, which showed the evidence of marked heterogeneity. Therefore subgroup analysis was done using study region and study design. As a result, the burden of pelvic organ prolapse was highest in Northern part of Ethiopia 99.2% whereas 88.7% in the study conducted with facility based setting **(**Table 2**).**

**Table 2:** Subgroup analyses on the burden of pelvic organ prolapse in Ethiopia, 2019)

Variables	Subgroup	No. of studies	Prevalence (95%CI)	I^2^ (%)	*P*-value
Sample design	Facility based	5	16.45 (12.12–20.78)	88.7	< 0.001
	community based	2	42.18 (39.91–44.46)	0	< 0.722
Study area	South East Ethiopia^a^	2	14.66 (11.02–18.31)	34	0.218
	Oromia	2	30.15 (10.39–49.92)	95.3	< 0.001
	Northern Ethiopia^b^	3	24.75 (2.67–46.83)	99.2	< 0.001

^a^ Addis Ababa & South nations nationalities and people, ^b^ Amhara & Tigray

### Factors associated with pelvic organ prolapse in Ethiopia

In this study, having body mass index (BMI) < 18.5, being rural resident and having age of the women > 40 years were the determinants factors of pelvic organ prolapse.

**Fig. 4:**
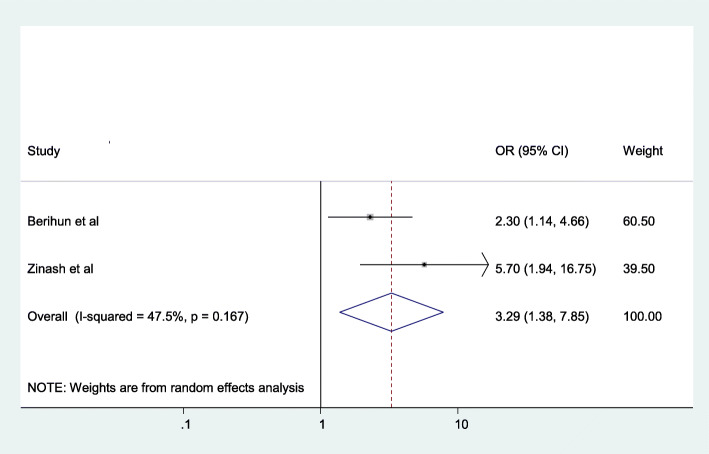
The association between resident and pelvic organ prolapse

The odds of having pelvic organ prolapse were (AOR = 3.29; 95% CI: 1.38–7.85), I^2^ = 47.5%, *P* = 0.167) 3.29 times prevalent among women who resided in rural areas than those who were living in urban areas **(**Fig. 4).

**Fig. 5:**
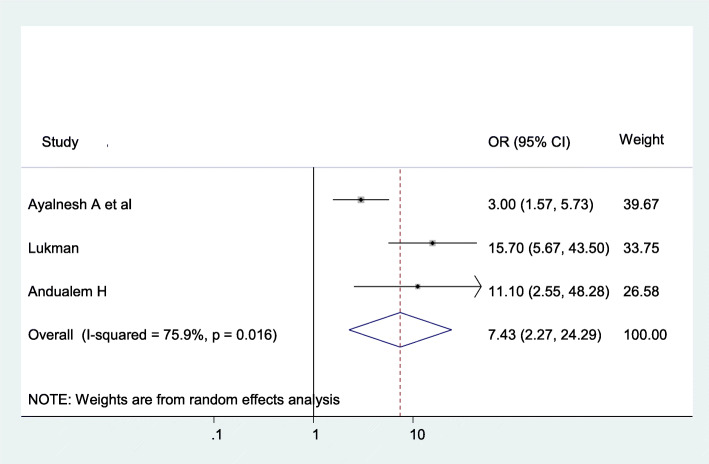
The association between Age and pelvic organ prolapse

The odds of having pelvic organ prolapse were 7.43 (AOR = 7.43; 95% CI: 2.27–24.29), I^2^ = 75.9%, *P* = 0.016) times more likely among women having more than 40 years old than the younger population **(**Fig. 5).

**Fig. 6:**
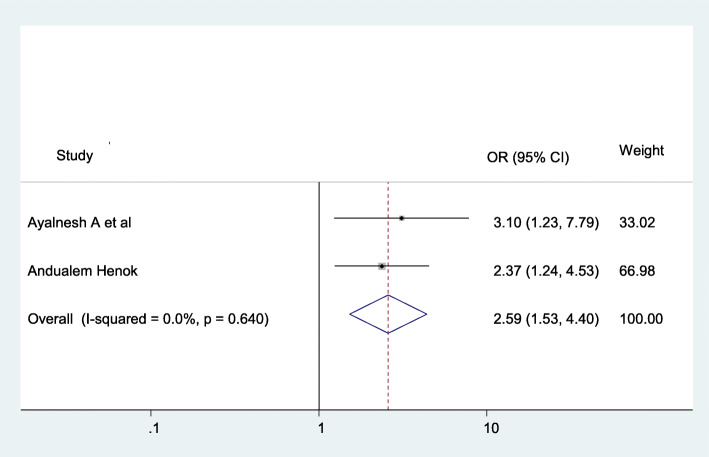
The association between BMI and pelvic organ prolapse

The chance of having pelvic organ prolapse were (AOR = 2.59; 95% CI: 1.53–4.4), I^2^ = 59.9%, *P* = 0.64), 2.59 times more likely among women who have less than 18.5 body mass index than the counterparts **(**Fig. 6).

## Discussion

In this meta-analysis, the national magnitude of pelvic organ prolpase in Ethiopia was 23.52% (95% CI: 14.3–32.74; I^2^ = 98.4%, *P* < 0.001). The finding of this study is consistent with the study done in China [28], accounts 2962 out of 20,008 women, estimated as 14.8%. This might be due to both of our study and the study conducted at China were involving large sample size as a result, increasing the sample size may increase the precision and the accuracy of the data.

The finding of this study is lower than the study done in Tanzania [29], and Gambia [30]. This highest magnitude might be justified as studies used different outcome measurements; such as, different methods of POP classification, inclusion of different age groups, and considering rural and urban resided women with different cultures and perceptions. Besides, this might be due to this systematic review and meta-analysis included studies done on nearly all institutional settings, as a result the tendency to face the burden in urban areas may be lower due to the availability and accessibility of health sectors, health professionals and mass media as a result the knowledge towards pelvic organ prolpase in the urban population may increase which leads to early detection of the problem and overcome the problem in urban resident populations, as a result the magnitude of POP is lower among women living in urban areas.

The prevalence of POP in this study is higher than the study done in Nepal [31], Nigeria [32], Ghana [33], and rural Pakistan [34]. This might be due to that this study constitutes the pooled result of different community and institutional based research findings.

Furthermore, in comparison with western countries the magnitude of POP in this systematic review and meta-analysis study was higher than reported magnitude among African American women. A study done in US the prevalence of POP was lower in African American women 1.9% as compared to white women 2.8% and Hispanic women 5.1% [29].

In this study, women residing in rural area (AOR = 3.29; 95% CI: 1.38–7.85) were 3.56 times more likely to have POP than the women living in urban areas. This might be justified by the burden of POP in African’s people living in United States of America and those residing in Africa comparatively higher number of home deliveries, difficult access to skilled delivery attendance, low attendance of institutional deliveries, lack of awareness on important risk factors, low coverage of family planning, lack of antenatal care follow up, lack of adequate postnatal care follow up with preventive measures, and heavier physical work load among people residing in living Africa predominantly in sub-Saharan Africa [4].

Age > 40 years (AOR = 7.43; 95% CI: 2.27–24.29) is the determinant factor to increase the risk of pelvic organ prolapse. This study finding is consistent with the study done in [35–37]. This might be due to the fact that the risk of POP increases with age because of due to pelvic muscle; ligaments and different pelvic structures are weakening as the age is increasing. As a result of advanced age, the physiological presenation of menopausal women are clearly seen. For example, kyphotic changes due to osteoporosis that developed secondary to advanced age and estrogen deficiency causes a horizontal shift in the pelvic brim which results in reflection of the abdominal contents to the pelvic floor and urogenital hiatus [38, 39].

Despite obesity (BMI 25 kg/m2) could increase the risk of POP, this systematic review and meta-analysis revealed a contrary finding that being underweight (BMI,18.5 kg/m2) increases the risk of POP threefold [40, 41]. On the contrary, this systematic review and meta-analysis finding showed that BMI ≤ 18 kg/m^2^ (AOR = 2.59; 95% CI: 1.53–4.4) is increasing the burden of pelvic organ prolapse. While weight gain is a risk factor for developing prolapse, weight loss does not come into sight to be significantly associated with the waning of POP, suggesting that damage to the pelvic floor related to weight gain might be irreversible [42].

Even though the original studies reported, high parity is one of the predictors of POP, estimating odds ratio is difficult since they simply reported the presence of association with chi square. However high parity (≥4) increases the risk of POP [33, 40]. This might be due to the fact that repeated pregnancy and birth damages sphincter muscles and ligaments, which sometimes never fully regain its strength and elasticity.

## Conclusion

The pooled prevalence of pelvic organ prolapse was higher. Residence, age and BMI were the predictors of pelvic organ prolpase. Identifying the modified and non modified risk factors for pelvic organ prolpase is a crucial strategic way to prevent further complications and risk of operation.

## Supplementary information

**Additional file 1: Table S1.** NOS Quality assessment tool for cross sectional and case control studies.

## Data Availability

The datasets used and/or analyzed during the current study are available from the corresponding author on reasonable request.

## Abbreviations

AOR: Adjusted Odds Ratio
ANC: Antenatal Care
EDHS: Ethiopia Demographic Health Survey
JBI: Joanna Briggs Institute
POP: Pelvic Organ Prolapse
WHO: World Health Organization
