# Supplementary material for: Burden of pelvic organ prolapse in Ethiopia: a systematic review and meta-analysis
Source: BMC Womens Health. 2020 Aug 6;20:166. doi: 10.1186/s12905-020-01039-w (PMC7412834; doi:10.1186/s12905-020-01039-w)
Supplement: Supplementary file 1 — Additional file 1: Table S1. NOS Quality assessment tool for cross sectional and case control studies. [file 12905_2020_1039_MOESM1_ESM.docx]

| **NOS scale for cross sectional studies** | Selection | | | | | | | | Comparability | | Outcome | | | | Total score | |
| --- | --- | --- | --- | --- | --- | --- | --- | --- | --- | --- | --- | --- | --- | --- | --- | --- |
|  | Representativeness  (1) | | Sample size  (1) | | Non-respondents  (1) | | Ascertainment of the exposure (risk factor)  (2) | | The subjects in different outcome groups are comparable, based on the study design or analysis. Confounding factors are controlled (2) | | Assessment of the outcome  (2) | | Statistical test  (1) | |  | |
| Tsegay B [11] | * | | * | | * | | ** | | * | | ** | | * | | 9 | |
| AndualemHenok [13] | * | | * | | * | | ** | | * | | ** | | * | | 9 | |
| Berihun et al [14] | * | | * | | * | | * | | * | | ** | | * | | 8 | |
| Dheresa et al [15] | * | | * | | * | | ** | | * | | ** | | * | | 9 | |
| *Menur A. et al* [16] | * | | * | | * | | ** | | * | | ** | | * | | 9 | |
| Lukman [17] | * | | * | | * | | ** | | * | | ** | | * | | 9 | |
| Lukman [17] | * | | * | | * | | ** | | * | | ** | | * | | 9 | |
| **NOS scale for case control studies** | Selection | | | | | | | Comparability | | Exposure | | | | | | Total score |
|  | Adequacy of case definition | Representativeness | | Selection of controls | | Definition of controls | | Comparability of cases and controls on the basis of the design or analysis | | Assessment of the exposure | | Same method of ascertainment for cases and controls | | Non-Response rate | |  |
| Zinash et al [18] | * | * | | * | | * | | ** | | * | | * | | * | | 9 |
| Asresie et al [12] | * | * | | * | | * | | ** | | * | | * | | * | | 9 |
